# Co-Immunoprecipitation Reveals Interactions Between Amelogenin and Ameloblastin *via* Their Self-Assembly Domains

**DOI:** 10.3389/fphys.2020.622086

**Published:** 2020-12-23

**Authors:** Rucha Arun Bapat, Jingtan Su, Janet Moradian-Oldak

**Affiliations:** Center for Craniofacial Molecular Biology, Herman Ostrow School of Dentistry, University of Southern California, Los Angeles, CA, United States

**Keywords:** enamel biomineralization, amelogenin (Amel), ameloblastin (Ambn), protein co-assembly, co-immunoprecipitation

## Abstract

Macromolecular assembly of extracellular enamel matrix proteins (EMPs) is intimately associated with the nucleation, growth, and maturation of highly organized hydroxyapatite crystals giving rise to healthy dental enamel. Although the colocalization of two of the most abundant EMPs amelogenin (Amel) and ameloblastin (Ambn) in molar enamel has been established, the evidence toward their interaction is scarce. We used co-immunoprecipitation (co-IP) to show evidence of direct molecular interactions between recombinant and native Amel and Ambn. Ambn fragments containing Y/F-x-x-Y/L/F-x-Y/F self-assembly motif were isolated from the co-IP column and characterized by mass spectroscopy. We used recombinant Ambn (rAmbn) mutants with deletion of exons 5 and 6 as well as Ambn derived synthetic peptides to demonstrate that Ambn binds to Amel *via* its previously identified Y/F-x-x-Y/L/F-x-Y/F self-assembly motif at the N-terminus of its exon 5 encoded region. Using an N-terminal specific anti-Ambn antibody, we showed that Ambn N-terminal fragments colocalized with Amel from secretory to maturation stages of enamel formation in a single section of developing mouse incisor, and closely followed mineral patterns in enamel rod interrod architecture. We conclude that Ambn self-assembly motif is involved in its interaction with Amel in solution and that colocalization between the two proteins persists from secretory to maturation stages of amelogenesis. Our *in vitro* and *in situ* data support the notion that Amel and Ambn may form heteromolecular assemblies that may perform important physiological roles during enamel formation.

## Introduction

Dental enamel formation involves a precisely orchestrated series of events in which assembly of extracellular enamel matrix proteins (EMPs) guides the formation of organized hydroxyapatite crystals (Margolis et al., 2006). Amelogenin (Amel) and ameloblastin (Ambn) are integral constituents of the forming enamel extracellular matrix. *In vivo* and *in vitro* studies reported that Amel is involved in regulating enamel thickness, controlling calcium phosphate mineral phase, and maintaining hydroxyapatite crystal growth and organization (Gibson et al., 2001; Fang et al., 2011; Bai et al., 2020), while Ambn was suggested to be involved in maintaining rod-interrod architecture (Paine et al., 2003), and ameloblast cell function (Fukumoto et al., 2004). Both proteins are intrinsically disordered and have strong tendency to self-assemble *in vitro* under a variety of solution conditions (Moradian-Oldak, 2012; Wald et al., 2013). Self-assembly domains within Amel and Ambn sequences have been previously identified and mutations in those domains affect enamel formation in mutant mouse models (Paine et al., 2002; Wald et al., 2017). Amel self-assembly is driven by two domains at its N‐ and C-termini, called as “domain A” and “domain B” respectively (Paine and Snead, 1997; Moradian-Oldak et al., 2000), whereas a Y/F-x-x-Y/L/F-x-Y/F motif located at the N-terminus of its exon 5 encoded region was identified for the self-assembly of Ambn (Wald et al., 2017).

Previous *in vitro* and *in situ* investigations have suggested molecular interactions and cooperative functions between EMPs during enamel crystal nucleation and maturation (Fan et al., 2011; Gallon et al., 2013). Specifically, it has been proposed that Amel and Ambn may function cooperatively to control some of the critical steps in the formation of enamel prismatic structure (Hatakeyama et al., 2009; Mazumder et al., 2016). Preliminary evidence that hints at interaction between Amel and Ambn begins at the secretory stage of enamel formation when these proteins are co-secreted through the same vesicles (Zalzal et al., 2008), and continues into maturation stage when their N-terminal fragments colocalize around molar enamel rods (Mazumder et al., 2014, 2016). Using surface plasmon resonance, Wald et al. (2017) suggested that Ambn self-assembly motif (YSRLGF motif in the mouse Ambn sequence) might play a role in binding to Amel but direct interactions between these two motifs were not demonstrated in solution.

Here, we provide direct evidence of the involvement of this conserved self-assembly domain in Amel-Ambn interaction using co-immunoprecipitation (co-IP). Recombinant Ambn (rAmbn) mutants with deletion of the sequences encoded by exons 5 and 6, Ambn-derived synthetic peptides representing the sequences encoded by exons 5 and 6, as well as an Ambn exon 5 peptide with a mutations in the YSRLGF self-assembly motif were used to confirm that this domain is indeed essential for binding of Ambn with Amel. Using mass spectrometry (MS) after co-IP of these two proteins from native porcine extract, we isolated Ambn fragments containing Y/F-x-x-Y/L/F-x-Y/F, confirming our findings from recombinant protein co-IP. *In situ* co-localization of Amel and N-terminal fragments of Ambn was analyzed from secretory to maturation stages in post-natal-day 8 (P8) wild-type mouse incisor enamel using immunohistochemistry. Longitudinal and transverse sectioning orientations were used to analyze the co-localization within the rod-interrod architecture. Our data confirm that the highly conserved self-assembly motif of Ambn can directly interact with Amel. We further demonstrate that colocalization of both proteins starts at the secretory stage and persists throughout the maturation stage.

## Materials and Methods

### Recombinant Protein Expression and Purification

Recombinant mouse Amel (rAmel) was expressed in BL21 *Escherichia coli* following published protocols and was precipitated by using saturated ammonium sulfate. The pallet was dissolved in 0.1% trifluoro acetic acid, and rAmel was purified by reversed phase HPLC (Simmer et al., 1994). Recombinant mouse Ambn (rAmbn) and rAmbn mutants with deletion of exons 5 or 6 (rAmbnΔ5 and rAmbnΔ6) were similarly expressed in BL21 *E. coli*. Protein from lysed *E. coli* was first concentrated by Ni-NTA column (QIAgen), and then dialyzed through a 10,000 Da dialysis membrane against ice cold phosphate buffer. Thioredoxin tag, S-tag, and histidine tag were cleaved by enzyme Enterokinase (light chain, New England Biolabs) at 37°C and rAmbn was purified by reversed phase HPLC (Su et al., 2019a,b). Purified rAmel and rAmbn proteins were characterized by sodium dodecyl sulfate polyacrylamide gel electrophoresis (SDS-PAGE) and mass spectrometry (Supplementary Figure 1). Proteins were lyophilized and stored at −20°C until further use.

### Porcine Enamel Matrix Protein Extraction

To obtain porcine EMPs, 6 month-old pig mandibles were purchased from Sierra for Medical Science (Whittier, CA, United States). Un-erupted second molars were extracted from their bony cavities, and EMPs were extracted from the crowns of the developing molars using previously published protocol (Uchida et al., 1995). Briefly, molar crowns were cleaned with ice cold phosphate buffered saline (PBS, pH 7.4) and newly formed enamel was scraped with a sharp razor blade on clean glass plates. Enamel scrapings were stirred overnight at 4°C in 0.5 M acetic acid. The slurry was desalted using Amicon Ultra 15 centrifugal filters to remove calcium phosphate. The supernatant was lyophilized and proteins were characterized using Western blots to confirm the presence of Amel and Ambn fragments (Supplementary Figure 2).

### Ambn-Derived Synthetic Peptides

Synthetic peptides derived from mouse Ambn protein regions encoded by exons 5 and 6 are referred here as Ambn-exon 5 derived peptide (AB2) and Ambn-exon 6 derived peptide (AB4), respectively (Su et al., 2016). AB2 was further divided into N‐ and C-terminal peptides peptide derived from N-terminus of AB2 (AB2N) and peptide derived from C-terminus of AB2 (AB2C; Chempeptide Ltd., China). A mutant of AB2N was designed by replacing the key amino acids in the YSRLGF motif with glycine, making it GSRGGG (Wald et al., 2017), which we refer to as peptide AB2N with mutation p.Y67G_L70G_F72G (AB2N-GGG; Biomertech, United States; Supplementary Figures 3, 4; Supplementary Table 1).

### Co-Immunoprecipitation

Co-immunoprecipitation protocol (Supplementary Scheme 1) was modified from Fan et al. (2009) and performed using a Pierce co-IP kit (Thermofisher Scientific). Manufacturer’s protocol was followed to bind either 10 μg anti-Amel (gift from Dr. Malcolm Snead) or 10 μg anti-Ambn antibody (AF3026, R&D systems) to the antibody coupling resin. Ten micrograms bait protein was added to the respective antibody bound column and incubated overnight at 4°C with gentle shaking. Columns were washed 5–6 times with Dulbecco’s PBS and 10 μg prey protein was added to the columns and bound for 4 h at 4°C. In case of native porcine EMP extract, the bait and prey proteins were loaded in the column as a mixture of all enamel matrix proteins. The bait-prey complex was eluted using 60 μl elution buffer provided with the kit. Control co-IP experiments were conducted in the absence of an antibody to visualize nonspecific binding to the column. Elution fractions were lyophilized and analyzed using SDS-PAGE, Western blots, and mass spectrometry.

### Mass Spectrometry (MS)

Elution fractions of native porcine EMP co-IP were analyzed by mass spectrometry at Scripps Center for Metabolomics and Mass Spectrometry (San Diego, CA, United States). Detailed protocol is described in Supplementary Material. Briefly, MS analysis was performed using nanoelectrospray capillary column packed with Zorbax SB-C18 stationary phase (Agilent). MS/MS data were obtained with an LTQ linear ion trap mass spectrometer using a nanoelectrospray source at 2 kV at the tip. All MS/MS data were searched against the NCBI Mammalia (mammals) database using Mascot (version 2.3.02; Matrix Science, London, United Kingdom). Proteins with a *p* < 0.05 (corresponding to a Mascot ion score greater than 57) were identified with two or more peptides and considered at 95% confidence level.

### Immunohistochemical Labeling of Post-Natal-Day 8 Mouse Incisors

Post-natal-day 8 wild-type C57BL/6 mice were euthanized following Institutional Animal Care and Use Committee (IACUC) protocols of the University of Southern California. Their mandibles were dissected, fixed (4% paraformaldehyde), decalcified (10% EDTA with 0.1% glutaraldehyde), paraffin embedded, and sectioned into 7 μm thick sections along the sagittal plane and anteroposteriorly along the transverse plane, maintaining the integrity of the developing incisor. Sections were immunohistochemically labeled with anti-Amel (gift from Dr. Malcolm Snead) or anti-Ambn (N-18, sc-33100, Santa Cruz Biotech, discontinued) primary antibodies (dilutions in Supplementary Table 2) and corresponding secondary antibodies conjugated with FITC or Alexa 488 for Amel and TRITC for Ambn using previously published protocol (Bapat and Moradian-Oldak, 2019). The slides were examined using a Leica SP-8 confocal microscope and data were analyzed with Leica Application Suite LAS-X version 1.8.1.13759. Manders’ colocalization coefficients (MCCs; Manders et al., 1993; Gallon et al., 2013) were utilized to quantify the colocalization between Amel and Ambn and calculated using Microsoft Excel. The change in colocalization coefficients between secretory and transition stage was calculated using a two-sample *t*-test, whereas the change in MCC values between secretory and maturation stages was calculated by Mann Whitney U-test in OriginPro data analysis software.

### Alizarin Red S Staining

Anteroposterior sections of P8 mouse incisor were deparaffinized using decreasing concentrations of xylene and ethanol, stained in freshly prepared 2% (w/v) Alizarin red S solution (pH 4.1–4.3) for 2–3 min, dehydrated in acetone followed by acetone-xylene solution (1:1), cleared in xylene, and mounted with a synthetic mounting medium (Dahl, 1952). Sections were observed using a Keyence BZX-810 microscope in bright-field mode.

## Results

### Recombinant Amel and Ambn Bind *in vitro*

**Figure 1:**
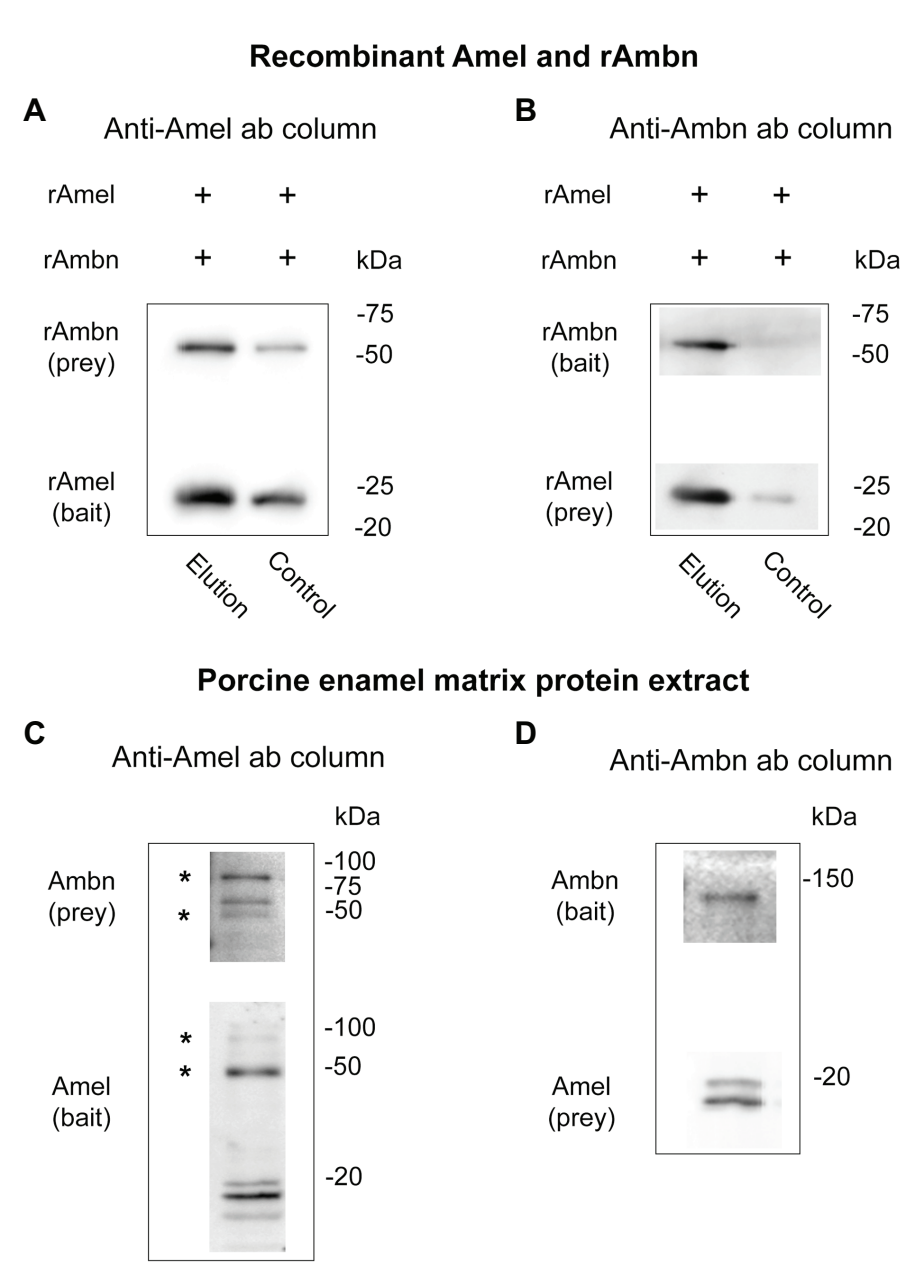
**(A–D)** Western blots showing **(A,B)** co-immunoprecipitation (co-IP) of recombinant Amel and Ambn; **(A)** lane 1 (elution) – co-IP of recombinant Ambn (rAmbn) with recombinant mouse Amel (rAmel) using rAmel as bait, lane 2 (control) – non-specific binding in the absence of an antibody is at least 50% less than elution; **(B)** lane 1 (elution) – co-IP of rAmel with rAmbn using rAmbn as bait, lane 2 (control) – no significant non-specific binding observed in the absence of antibody; **(C,D)** co-IP of native Amel and Ambn from porcine enamel matrix protein extract using **(C)** anti-Amel antibody column, **(D)** using anti-Ambn antibody column; asterisks (^*^) in **(C)** mark putative Amel-Ambn complexes.

Direct binding between rAmel and rAmbn was confirmed by co-IP using anti-Amel and anti-Ambn antibodies. rAmbn bound to rAmel bait (Figure 1A, elution), and rAmel bound to rAmbn bait (Figure 1B, elution) to co-elute from the co-IP columns. Control experiment in the absence of anti-Amel antibody showed ~50% less rAmbn and rAmbn bound to the column (Figure 1A, control) and in the absence of anti-Ambn antibody no significant non-specific binding to the co-IP resin was observed (Figure 1B, control).

### Native Amel-Ambn Complexes Co-Elute From Porcine EMP Extract

Support for direct binding between native Amel and Ambn proteins was obtained by their co-IP from porcine second molar EMP extract (Figures 1C,D). Three bands at ~85, ~65, and ~45 kDa were labeled by the anti-Ambn antibody in the elution fractions of native EMP co-IP when anti-Amel antibody was used for the co-IP column (Figure 1C). Interestingly, the same 85 and 45 kDa bands were also labeled in anti-Amel Western blot (Figure 1C, asterisks). In the same blot, native Amel fragments were identified as previously described “18,” “20,” and central “13 k” fragments (Ryu et al., 1999; Figure 1C). Mass spectra from the elution fractions of this co-IP revealed Ambn peptides from exons 4 and 5 encoded region containing a partial Y/F-x-x-Y/L/F-x-Y/F motif with a protein score > 57, corresponding to 95% confidence level (Supplementary Table 3). The interaction was confirmed by reversing the experiment with anti-Ambn antibody in the co-IP column, which revealed Ambn in a single band at ~130 kDa, and Amel as 18 and 20 kDa bands (Figure 1D). Mass spectrometric analysis of this co-IP identified Ambn peptides matching the exons 3–5 encoded fragments, containing the entire Y/F-x-x-Y/L/F-x-Y/F motif (95% confidence level). Mass spectrometric analysis of co-IP elution fractions from both experiments also identified Amel peptides located within tyrosine rich amelogenin polypeptide (TRAP) containing the Y/F-x-x-Y/L/F-x-Y/F self-assembly motif but with confidence level < 95% (Supplementary Table 3).

### YSRLGF Motif of Ambn is Essential for Binding With Amel

**Figure 2:**
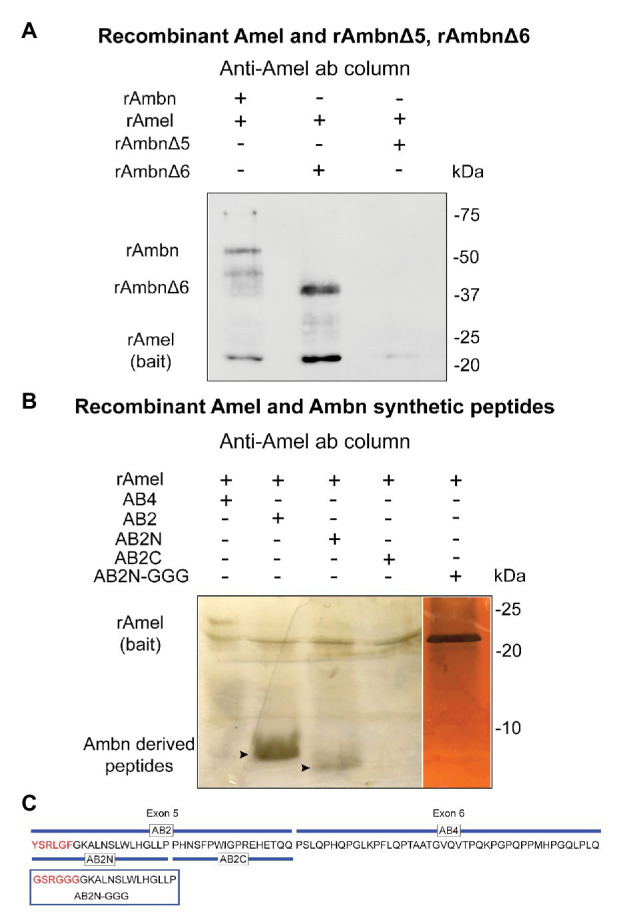
**(A)** Western blot showing lane 1 – rAmel and rAmbn co-IP positive control, lane 2 – rAmbnΔ6 co-elutes with rAmel, and lane 3 – rAmbnΔ5 fails to bind to rAmel; **(B)** 16% sodium dodecyl sulfate (SDS) gels stained with silver stain showing lane 1 – Ambn-exon 6 derived peptide (AB4) did not bind to rAmel, lane 2 – Ambn-exon 5 derived peptide (AB2) co-elutes with rAmel (arrowhead), lane 3 – peptide derived from N-terminus of AB2 (AB2N) containing the self-assembly motif YSRLGF co-elutes with rAmel (arrowhead), lane 4 – peptide derived from C-terminus of AB2 (AB2C) did not bind to rAmel, and lane 5 – peptide AB2N (AB2N-GGG) with mutated YSRLGF motif (p.Y67G_L70G_F72G) did not bind to rAmel; **(C)** schematic representation of mouse Ambn sequence encoded by exons 5 and 6 showing Ambn derived synthetic peptides AB2, AB2N, AB2C, AB4, and mutant peptide AB2N-GGG.

In order to confirm the region of rAmbn that interacts with rAmel, co-IP experiments were repeated using mutants rAmbnΔ5 and rAmbnΔ6. Co-IP between rAmel and rAmbn was used as a positive control (Figure 2A, lane 1). Mutant rAmbnΔ6 retained its ability to bind to Amel and co-eluted with Amel (Figure 2A, lane 2); however, rAmbnΔ5 lost its ability to bind to Amel and did not co-elute (Figure 2A, lane 3); suggesting that exon 5 encoded region of Ambn is essential for its interaction with Amel. Co-IP of rAmel with Ambn exon 5 derived synthetic peptides AB2 and AB2N containing the YSRLGF motif confirmed the direct binding of this region to rAmel (Figure 2B, lanes 2 and 3, respectively). Peptide AB2C encoding the C-terminus of exon 5 lacking the YSRLGF motif did not bind to rAmel (Figure 2B, lane 4). Peptide AB4 representing Ambn exon 6 region also failed to bind to rAmel (Figure 2B, lane 1). Ambn peptide AB2N-GGG with YSRLGF motif mutated to GSRGGG also lost its ability to bind to rAmel (Figure 2B, lane 5). The sequences of Ambn synthetic peptides and mutant peptide AB2N-GGG are shown in Figure 2C. Masses of peptides are listed in Supplementary Table 1.

### Amel-Ambn Co-Localization at Different Stages of Amelogenesis in Developing Mouse Incisor

**Figure 3:**
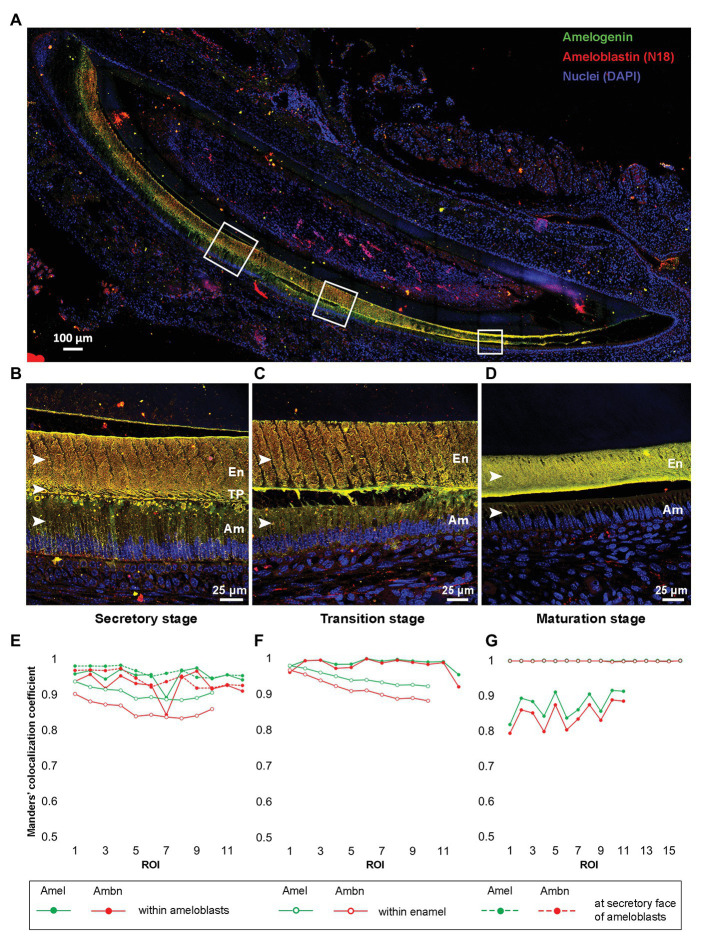
**(A)** Merged tile-scan confocal image of a longitudinal section from post-natal-day 8 (P8) mouse incisor co-labeled with anti-Amel (green) and anti-Ambn N-18 (red) antibodies. **(B–D)** Maximum intensity projections of Z-stacks of secretory, transition, and maturation stage areas marked by white squares in **(A)**; **(E–G)** Manders’ colocalization coefficients (MCCs) for Amel and Ambn at different regions within secretory, transition, and maturation stages of enamel formation, respectively. **(B,E)** secretory stage, MCC calculated within enamel matrix, at secretory face of ameloblasts, and within ameloblasts (white arrowheads in **B**); **(C,F)** transition stage, MCC calculated within enamel matrix and inside ameloblasts (white arrowheads in **C**) and **(D,G)** maturation stage, MCC calculated within enamel matrix and within ameloblasts (white arrowhead in **D**). En, enamel; TP, Tomes’ processes; and Am, ameloblasts.

Tile-scan image of P8 incisor showed the presence of Amel and N-18 antibody labeled Ambn within ameloblasts (Am), at the secretory fronts of ameloblasts, and throughout the entire bulk of the enamel thickness from secretory to maturation stage of enamel formation (Figure 3A). At a higher resolution, the maximum intensity projections of Z-stacks of secretory, transition, and maturation stages (Figure 3B-D) of enamel formation clearly depicted Amel-Ambn colocalization. In secretory stage (Figure 3B), the MCC for Amel was 0.95 and for Ambn 0.92 within ameloblasts. The values were similar to the MCC within secretory stage Tomes’ processes (TP), 0.96 for Amel, and 0.94 for Ambn (Figure 3E). In transition stage (Figure 3C), the MCC for both Amel and Ambn within ameloblasts increased to 0.98 (Figure 3F) but decreased as maturation progressed to 0.87 for Amel and 0.84 for Ambn (Figures 3D,G). Within the enamel matrix in secretory stage (Figure 3B), the MCC for Amel was 0.90 and for Ambn 0.85 (Figure 3E), meaning about 90% of labeled Amel colocalized with 85% of labeled Ambn fragments. In the transition stage enamel matrix (Figure 3C), the MCC values for Amel and Ambn increased to 0.94 and 0.91, respectively (Figure 3F), finally culminating at almost 0.99 for both Amel and Ambn in maturation stage enamel (Figures 3D,G). The Amel and Ambn colocalization within enamel matrix increased significantly from secretory to maturation stages (*p* < 0.001). The N-18 antibody labeled full-length Ambn, along with proteolytic cleavage products of Ambn. Based on N-18 antibody epitope, these fragments potentially contained regions in close proximity to the exon 5 encoded region (Supplementary Figure 3). For comparison, M300 antibody detected Ambn only within ameloblasts and at the Tomes’ processes but did not label Ambn proteolytic cleavage fragments within the bulk of the enamel matrix (Supplementary Figure 5).

**Figure 4:**
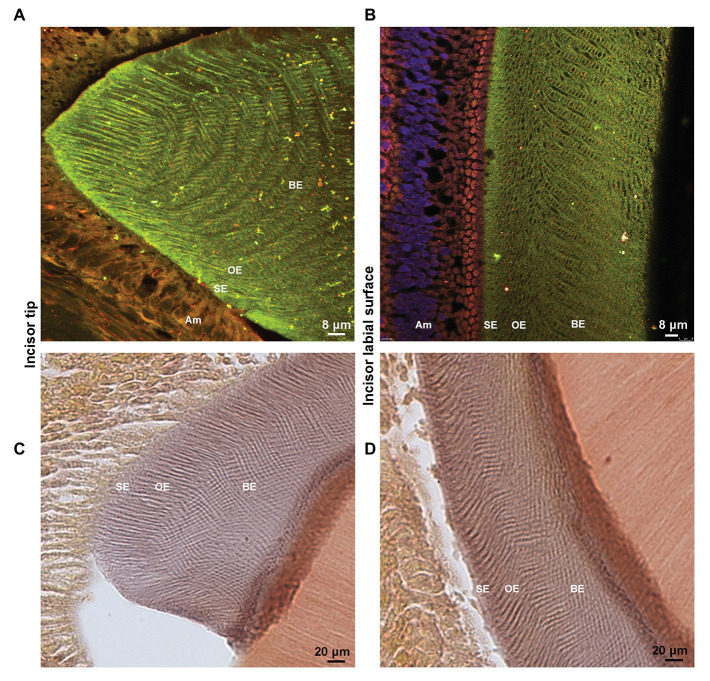
**(A)** Maximum projections of Z-stack confocal images and **(B)** a single confocal image of an anteroposterior section from maturation-stage P8 enamel showing co-localization of Amel (green) and Ambn (red, N18 antibody) and the rod-interrod architecture. **(C,D)** Bright field image of Alizarin red S stained P8 incisor sections depicting residual mineral in maturation stage enamel rod-interrod architecture. **(A,C)** tip of the incisor **(B,D)** labial surface of the incisor. Am, ameloblasts; SE, surface enamel; OE, outer enamel; and BE, bulk enamel.

To confirm that Amel-Ambn co-localization persists during the maturation stage and to provide better visualization, anteroposterior sections through the tip of the incisor were used. Clearly demarcated enamel rod architecture was visible due to Amel co-localizing with N-terminal-containing fragments of Ambn in the maturing enamel (Figure 4A). Distal to the tip of the incisor, Amel-Ambn colocalization could be differentiated into surface enamel (SE), outer enamel (OE), and bulk enamel (BE) layers based on the colocalization pattern (Figure 4B). Alizarin Red S staining of demineralized sections revealed that there was residual calcium present in the maturation stage enamel even after EDTA demineralization (Figures 4C,D). The pattern of Amel-Ambn co-assembly in the anteroposterior sections closely mimicked the pattern of the mineral remnants and the rod-interrod structure observed previously in SEM images of non-demineralized mouse enamel (Lacruz et al., 2012).

## Discussion

The essential physiological functions of both Amel and Ambn proteins in the formation of normal enamel have been documented in knockout and mutant animal models (Gibson et al., 2001; Paine et al., 2002; Fukumoto et al., 2004; Liang et al., 2019). Mutations in *AMELX* or *AMBN* genes in humans disrupt enamel formation and cause *Amelogenesis Imperfecta* (AI) with a range of clinical presentations including but not limited to very thin mineralized enamel or soft friable enamel (hypoplastic or hypomineralized, respectively; Smith et al., 2017).

*In vitro* and *in vivo* studies have suggested that protein assembly is the principal mechanism for controlling nucleation, growth, and organization of mineral crystals during enamel biomineralization (Margolis et al., 2006; Moradian-Oldak, 2012). Enamel extracellular proteins may function in a cooperative or synergic manner and direct interactions or co-assembly may be a requirement for their role (Fan et al., 2011; Wald et al., 2017; Tao et al., 2018).

The critical role of the Y/F-x-x-Y/L/F-x-Y/F motif at the N-terminus of Ambn exon 5-encoded region was recently identified in Ambn self-assembly (Wald et al., 2013, 2017). A mouse model having key residues of this domain mutated to glycine showed a lack of patterning in secreted Amel matrix leading to disorganized enamel crystals (Wald et al., 2017). The authors suggested that the Y/F-x-x-Y/L/F-x-Y/F motif may be involved in Amel-Ambn co-assembly along with Ambn self-assembly. The results of our current study show direct evidence of such interactions in solution, and our findings support that the abnormal enamel formation observed in the mutant animals by Wald et al. (2017) could be a result of interruptions in Ambn-Amel interactions.

Using co-IP, we confirmed that rAmbn directly binds to rAmel in solution. Here, the occurrence of this interaction in *in vivo* extracts is established by co-IP of native Amel and Ambn proteins extracted from porcine enamel in developing second molars. This confirmed that the interaction is occurring *via* a region devoid of any post translational modifications (Kobayashi et al., 2007) as it remained consistent in recombinant (*E. coli* derived) and native proteins. The 85 and 45 kDa bands detected in native porcine co-IP experiments with anti-Ambn antibody in the column could not be ascribed to either native Amel or Ambn fragments described in literature (Ryu et al., 1999; Iwata et al., 2007). These could be putative Amel and Ambn complexes.

As documented in mutant animal models, *Ambn* exons 5 and 6 are key in Ambn protein function and are involved in maintaining ameloblast cells (Fukumoto et al., 2004; Wazen et al., 2009). We identified that Ambn mutants with deletion of exons 5 (rAmbnΔ5) did not bind to Amel but rAmbnΔ6 retained its ability to bind to Amel. This implied that the region encoded by exon 5 of *Ambn* is essential for its interaction with Amel. AB2, the synthetic peptide derived from sequence encoded by exon 5 bound to Amel. To narrow down the location of the binding domain within AB2, we employed peptides AB2N, AB2C, and AB2N-GGG representing the N‐ and C-termini of AB2 and a peptide with mutated Y/F-x-x-Y/L/F-x-Y/F motif, respectively. AB2 and AB2N bound to Amel but AB2C and AB2N-GGG did not, thus confirming that the Y/F-x-x-Y/L/F-x-Y/F motif of Ambn is involved in its interaction with Amel. An *AI* case with a mutation within exon 5 of *AMBN* has not yet been identified, but deletion of exon 6 encoded region of *AMBN* which is adjacent to Ambn self-assembly Y/F-x-x-Y/L/F-x-Y/F motif does cause *AI* in humans (Poulter et al., 2014).

Moreover, a proline to threonine mutation adjacent to one of the self-assembly motifs of Amel within the TRAP region has also been identified which leads to hypomaturation type of *AI* (Collier et al., 1997). These mutations could potentially affect Amel-Ambn interactions intensifying the *AI* phenotype. The two evolutionary conserved Y/F-x-x-Y/L/F-x-Y/F motifs are also present within the TRAP peptide of Amel which overlaps with it’s self-assembly “A domain” (Paine and Snead, 1997; Wald et al., 2017). Mutations in the “A domain” lead to disruption of protein-protein interaction and ultimately to enamel malformation (Moradian-Oldak et al., 2000; Paine et al., 2002). It has been previously shown that TRAP accumulates in maturation stage enamel (Fincham et al., 1981; Mazumder et al., 2016) and can directly interact with Ambn *in vitro* (Su et al., 2016). In this study, mass spectrometry identified Amel peptides from the TRAP region, including one containing the self-assembly motif in co-IP elution fractions of porcine enamel matrix protein extract. Our current observation together with previously published data of Amel self-assembly supports the conclusion that the self-assembly domains on both Ambn as well as Amel may function as the interacting domains between the two proteins.

We previously reported colocalization of Amel and Ambn N-terminal fragments within the sheath space of maturation stage molar enamel (Mazumder et al., 2016). In this study, the rationale behind demonstrating Amel-Ambn colocalization in the mouse incisor model was to compare and identify possible changes in their colocalization as enamel maturation progressed. The persistent significant increase in colocalization coefficient observed from secretory to maturation stage enamel in a single incisor suggested continued interaction between not only nascent Amel and Ambn but also their proteolytic cleavage products.

The function of Amel-Ambn interactions and their biological significance are important subjects of our current and future investigations. One such function maybe related to maintaining the ameloblast-enamel matrix interface. We recently showed that Ambn localizes at the ameloblast-enamel matrix interface and interacts with ameloblast cell membrane (Su et al., 2019b, 2020). Considering that Amel is the main component of the matrix, binding of Ambn with Amel and with ameloblast cell membrane may together maintain the ameloblast-enamel matrix interface. This could also explain the severe separation of ameloblasts from underlying matrix in mice with deleted *Ambn* exons 5 and 6 (Fukumoto et al., 2004). Another aspect of such interactions may be related to the synergic function of these proteins in controlling mineral nucleation and growth (Fan et al., 2011; Tao et al., 2018). The ability of Ambn C-terminal region to bind calcium has been well-documented (Yamakoshi et al., 2001; Tarasevich et al., 2007; Zhang et al., 2011) suggesting Ambn’s involvement in mineralization. A recent report of *AI* with a point mutation in the calcium-binding region of Ambn described enamel with low mineral density (Lu et al., 2018).

In conclusion, we provide direct evidence for Ambn and Amel protein interactions in solution and demonstrate their colocalization *in situ* in mouse incisor, starting at the secretory stage and persisting until maturation stage. Dissecting Amel-Ambn co-assemblies and exploring the details of Amel-Ambn molecular interactions in the presence of calcium are the subjects for future investigations.

## Data Availability Statement

The original contributions presented in the study are included in the article/Supplementary Material, further inquiries can be directed to the corresponding author.

## Ethics Statement

The animal study was reviewed and approved by University of Southern California IACUC.

## Author Contributions

RAB designed and performed the experiments, analyzed the data, and wrote the manuscript. JS helped with protein synthesis and peptide design. JM-O designed experiments, analyzed data, wrote and critically revised the manuscript. All authors contributed to the article and approved the submitted version.

### Conflict of Interest

The authors declare that the research was conducted in the absence of any commercial or financial relationships that could be construed as a potential conflict of interest.

## Abbreviations

AB2: Ambn-exon 5 derived peptide
AB2C: Peptide derived from C-terminus of AB2
AB2N: Peptide derived from N-terminus of AB2
AB2N: GGG, Peptide AB2N with mutation p.Y67G_L70G_F72G
AB4: Ambn-exon 6 derived peptide
AI: *Amelogenesis imperfecta*
Ambn/rAmbn: Ameloblastin/recombinant ameloblastin
AmbnΔ5: Ambn p.Y67_Q103del
AmbnΔ6: Ambn p.P104_V168del
Amel/rAmel: Amelogenin/recombinant amelogenin
Co-IP: Co-immunoprecipitation
EMP: Enamel matrix protein
MCC: Manders’ colocalization coefficient
SDS-PAGE: Sodium dodecyl sulfate polyacrylamide gel electrophoresis
TRAP: Tyrosine rich amelogenin polypeptide
