# Supplementary material for: Co-Immunoprecipitation Reveals Interactions Between Amelogenin and Ameloblastin via Their Self-Assembly Domains
Source: Front Physiol. 2020 Dec 23;11:622086. doi: 10.3389/fphys.2020.622086 (PMC7786100; doi:10.3389/fphys.2020.622086)
Supplement: Supplementary file 1 [file Table_1.docx]

# Co-immunoprecipitation Reveals Interactions between Amelogenin and Ameloblastin via their Self-Assembly Domains (Rucha Arun Bapat, Jingtan Su, Janet Moradian-Oldak)

# Supplementary Information


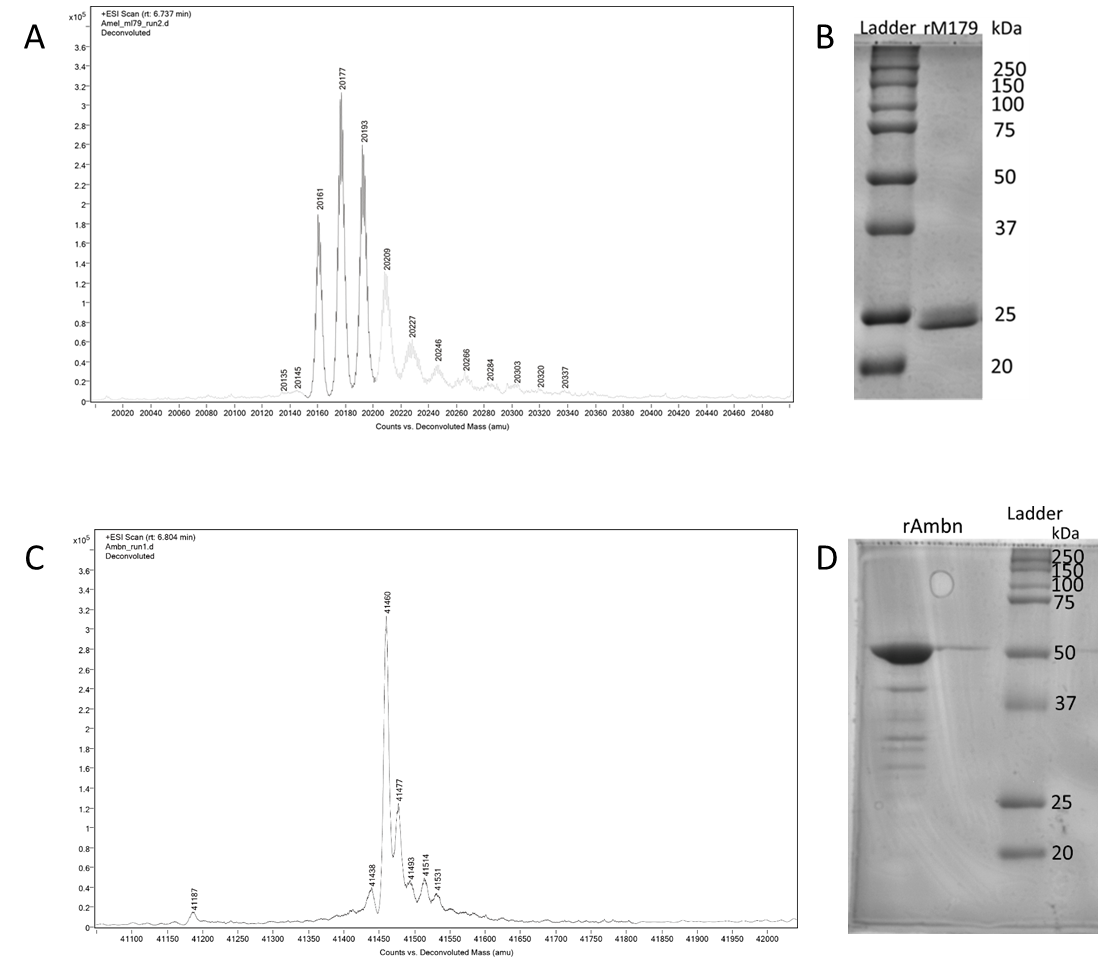


## Supplementary Fig 1. Characterization of rAmel (A&B) and rAmbn (C&D) by mass spectrometry (performed by Dr. Dmitry Eremin at Agilent Center for Excellence in Biomolecular Characterization USC) and SDS-PAGE.


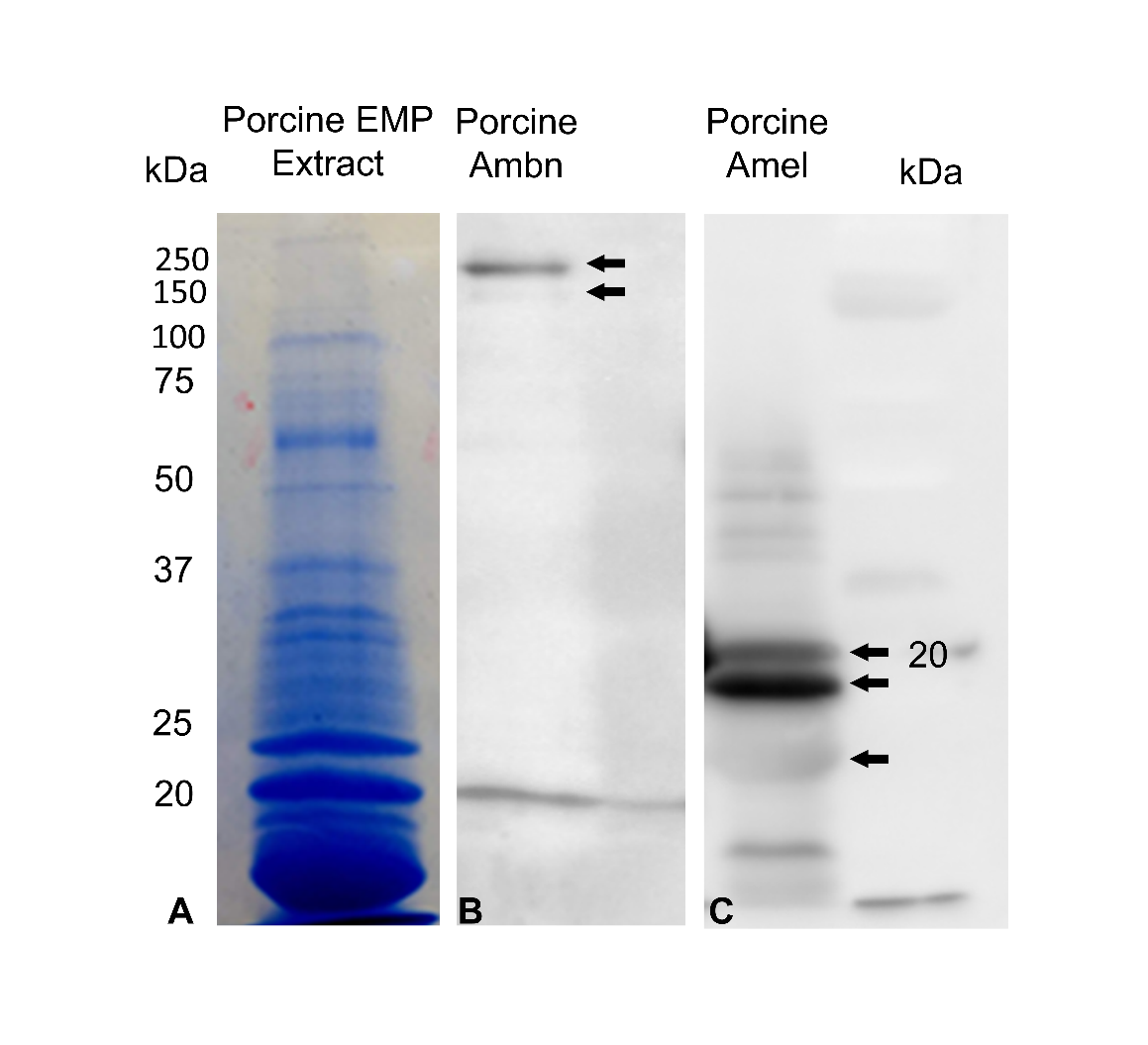


## Supplementary Fig 2. Characterization of porcine enamel matrix protein extract with SDS-PAGE (A) and Western blots (B&C). (A) 12% SDS gel stained with Coomassie blue showing all the detectable proteins and protein fragments from porcine EMP extract; (B) Western blot against Ambn showing porcine Ambn as 2 bands (arrows); (C) Western blot against Amel showing characteristic porcine Amel “20 k” and “18 k” bands along with 13 kDa central region (arrows).

**
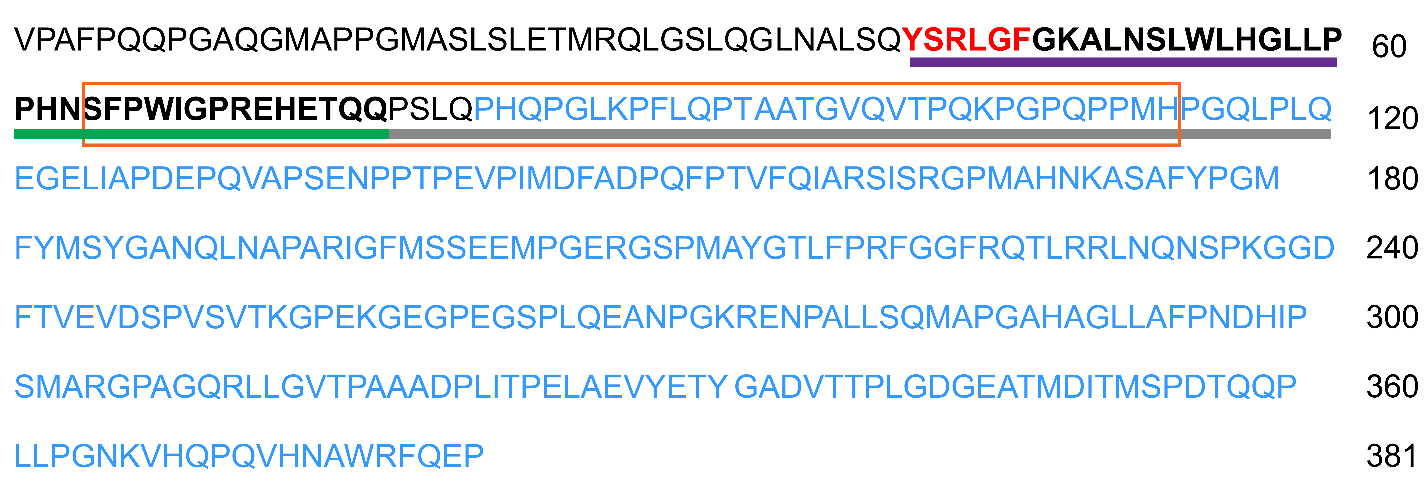
**

## Supplementary Fig 3. Mouse ameloblastin sequence (GenBank accession number AAB93765.1) depicting M300 antibody epitope in blue and the region of N-18 antibody epitope in the orange box. The exon 5 encoded region (peptide AB2) is in bold letters, YSRLGF self-assembly motif within exon 5 is indicated in bold red, peptide AB2N is underlined in purple, and peptide AB2C is underlined in green. Ambn mutant rAmbnΔ5 lacked region encoded by exon 5. The exon 6 encoded region (peptide AB4) has a grey underline and mutant rAmbnΔ6 lacked region encoded by exon 6.

##
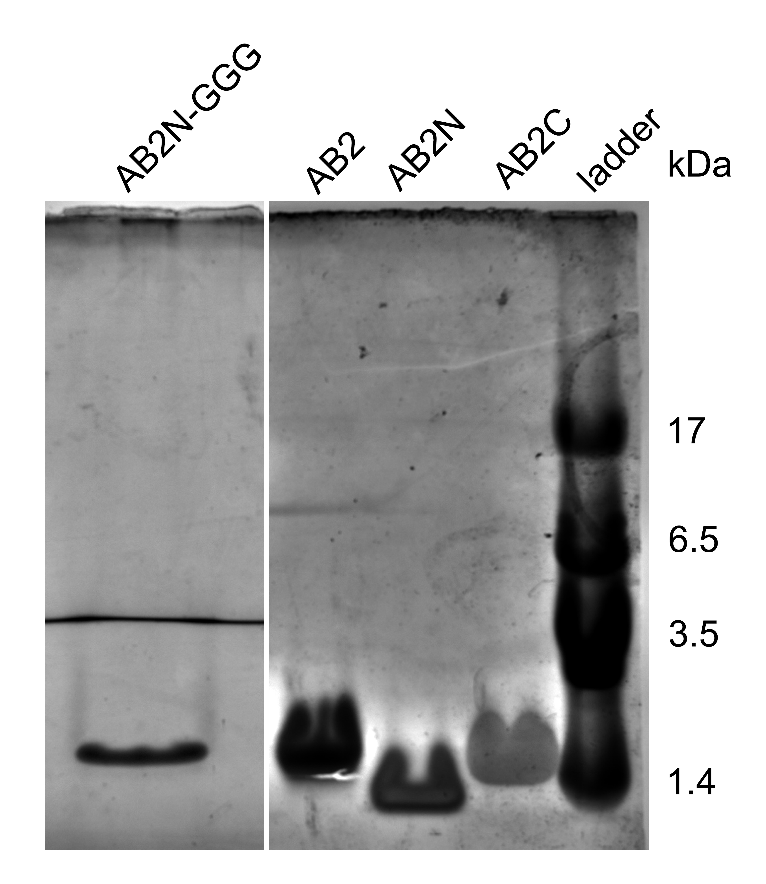
Supplementary Fig 4. Ambn synthetic peptides (AB2, AB2N, and AB2C) and mutant peptide (AB2N-GGG) characterized on a 16% SDS-PAGE gel, stained by silver staining.

**
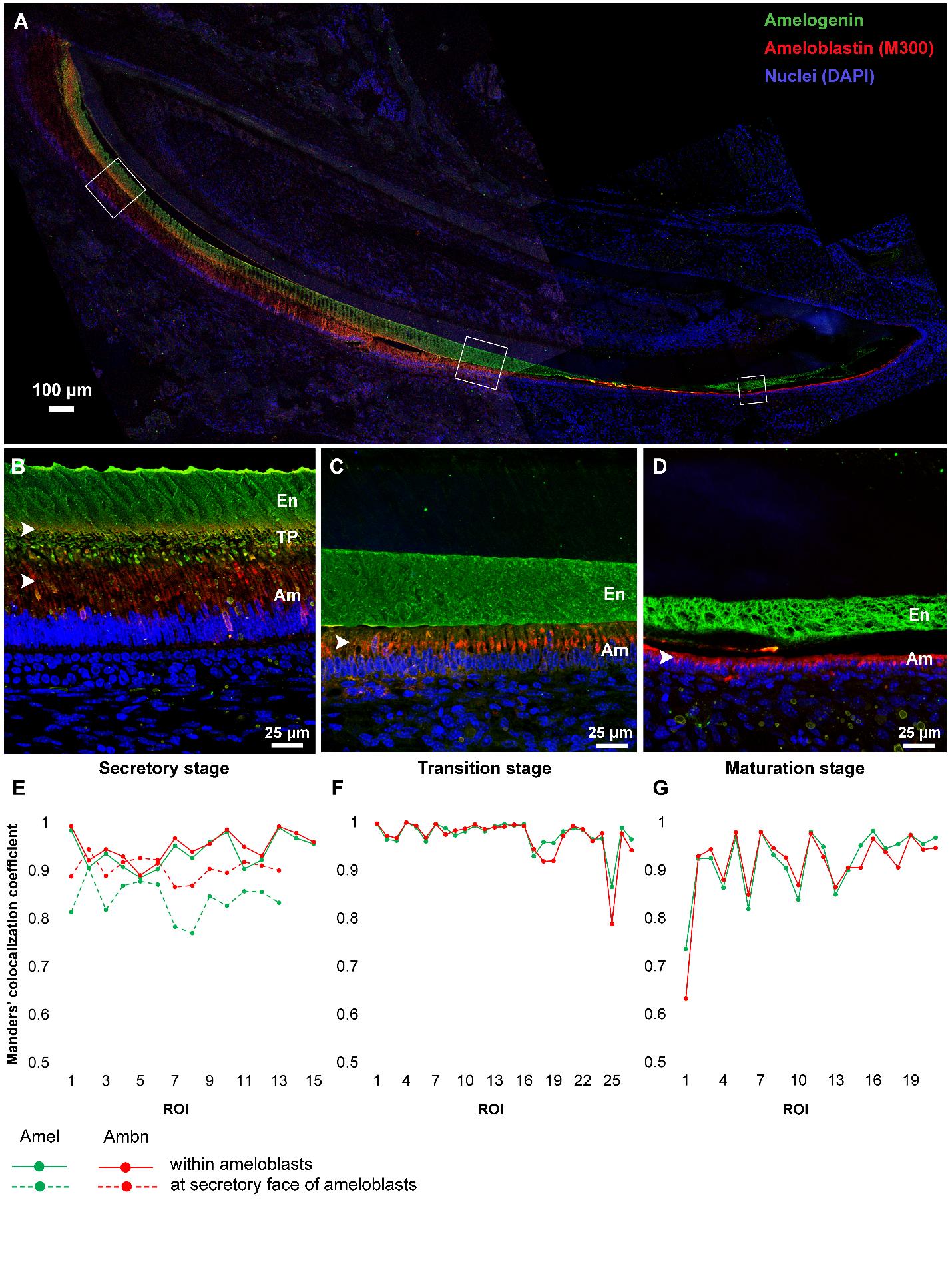
**

Supplementary Fig 5. (A) Merged tile-scan confocal image of a longitudinal section from P8 mouse incisor co-labeled with anti-Amel (green) and anti-Ambn M300 (red) antibodies; (B-D) Maximum intensity projections of Z-stacks of secretory, transition, and maturation stage areas marked by white squares in A; (E-G) Manders’ colocalization coefficients for Amel and Ambn at different regions within each stage of enamel formation. (B&E) secretory stage, MCC calculated within ameloblasts and at the secretory face of ameloblasts (white arrowheads in B); (C&F) transition stage, MCC calculated within ameloblasts (white arrowhead in C) and (D&G) maturation stage, MCC calculated within ameloblasts (white arrowhead in D). En- enamel, TP- Tomes’ processes, Am- ameloblasts.

## Supplementary Table 1. The amino acid sequences and masses of Ambn-derived synthetic peptides (AB2, AB2N, AB2C, AB4) and mutant peptide (AB2N-GGG).

| Peptide | Sequence | Exon | Mass (Da) |
| --- | --- | --- | --- |
| AB2 | ^41^YSRLGFGKALNSLWLHGLLPPHNSFPWIGPREHETQQ^77^ | 5 | 4284.89 |
| AB2N | ^41^YSRLGFGKALNSLWLHGLLP^61^ |  | 2242.65 |
| AB2C | ^62^PHNSFPWIGPREHETQQ^77^ |  | 2060.22 |
| AB2N-GGG | ^41^GSRGGGGKALNSLWLHGLLP^61^ |  | 1990.30 |
| AB4 | ^78^PSLQPHQPGLKPFLQPTAATGVQVTPQKPGPQPPMHPGQLPLQ^121^ | 6 | 4542.28 |

## Supplementary **Table 2.** Dilutions of Anti-Amel and anti-Ambn antibodies used in immunohistochemistry (IHC) co-immunoprecipitation (co-IP), and Western blots; NA: antibody was not used for the specified experiment.

| **Protein** | **Antibody** | **Host** | **Epitope** | **Concentration** | | |
| --- | --- | --- | --- | --- | --- | --- |
|  |  |  |  | **IHC** | **Co-IP column** | **Western blots** |
| Amelogenin | Custom antibody, gift from Dr. Malcolm Snead | Chicken | Polyclonal against the full-length protein. | 1:1000 | 10 μg | 1:1000 |
| Ameloblastin | N-18, sc-33100, Santa Cruz Biotech (discontinued) | Goat | Polyclonal, against a peptide mapping near the N-terminus (see Supplementary Fig 3) | 1:100 | NA | NA |
|  | M300, sc-50534, Santa Cruz Biotech (discontinued) | Rabbit | Polyclonal against 300 amino acids excluding the N-terminus (see Supplementary Fig 3) | 1:500 | NA | 1:1000 |
|  | Anti-ameloblastin ab, AF3026, R&D systems | Goat | Polyclonal, against the entire protein except the signal peptide. Epitope- valine27 to proline407 | NA | 10 μg | 1:1000 |

## Supplementary Table 3. Amel and Ambn peptide fragments collected from porcine EMP co-IP elution fractions and identified by mass spec. Peptides in white boxes have > 95% confidence level and those in gray boxes are < 95% confidence level.

| **Protein** | **Peptide** | **Region within the protein sequence** | **Expected mass (Da)** | **Calculated mass (Da)** | **Antibody in the co-IP column** |
| --- | --- | --- | --- | --- | --- |
| Ameloblastin | QPGTPGVASLSLETMR | Exons 3&4 | 1643.65 | 1642.83 | Anti-Amel  (Fig 1C) |
|  | QLGSLQGLNMLSQYSR | Exons 4&5 | 1794.55 | 1793.90 |  |
| Amelogenin | MPLPPHPGHPGYINFSYEK | Tyrosine rich amel polypeptide (TRAP) | 2180.90 | 2180.05 |  |
|  | WYQNMIR |  | 1011.03 | 1009.48 |  |
|  | | | | | |
| Ameloblastin | QPGTPGVASLSLETMR | Exons 3&4 | 1643.74 | 1642.83 | Anti-Ambn  (Fig 1D) |
|  | QLGSLQGLNMLSQYSR | Exons 4&5 | 1794.55 | 1793.90 |  |
|  | QPGTPGVASLSLETMRQLGSLQGLNMLSQYSR | Exons 3-5 | 3419.45 | 3418.73 |  |
|  | FGFGK | Exon 5 | 554.68 | 554.28 |  |
|  | SFNSLWMHGLLPPHSSFQWMRPR | Exon 5 | 2811.92 | 2810.36 |  |
| Amelogenin | PVLTPLKWYQNMIR | Tyrosine rich amel polypeptide (TRAP) | 1758.72 | 1757.96 |  |
|  | MPVLTPLKWYQNMIR |  | 1888.61 | 1889.00 |  |
|  | MPLPPHPGHPGYINFSYEK |  | 2181.08 | 2180.05 |  |
|  | WYQNMIR | Tyrosine rich amel polypeptide (TRAP) | 1010.12 | 1009.48 |  |


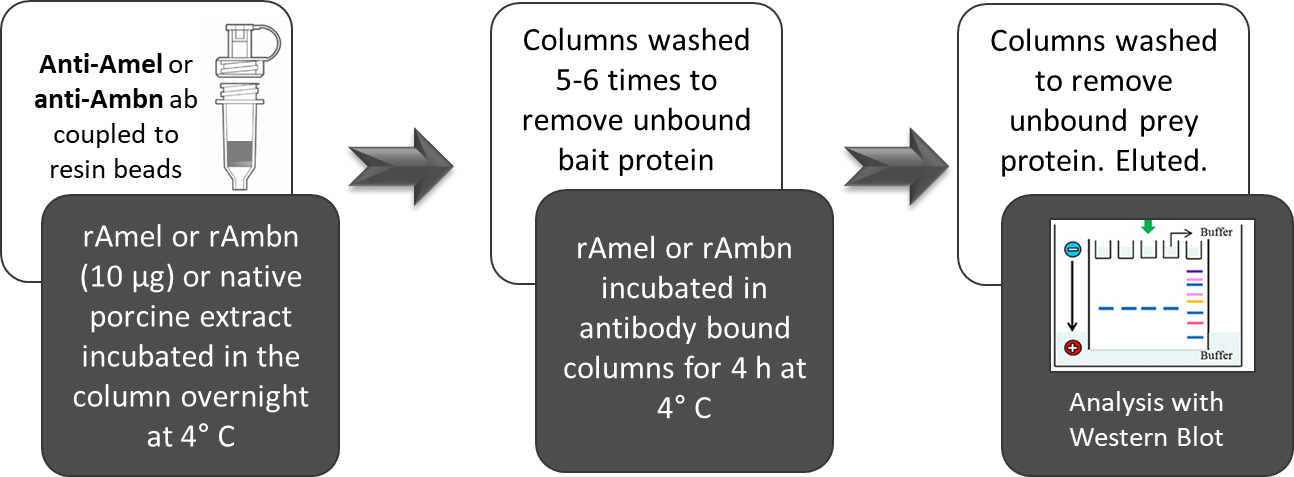
Supplementary Scheme 1. Schematic describing co-immunoprecipitation protocol followed to determine direct interaction between Amel and Ambn. Step of incubating rAmel or rAmbn prey protein for 4 h was skipped for native porcine extract which was loaded as a mixture of all proteins.

# Materials and Methods

## **Mass Spectrometry**

Elution fractions of native porcine EMP co-IP were analyzed by mass spectrometry at Scripps Center for Metabolomics and Mass Spectrometry (San Diego, CA). Samples were subjected to reverse-phase chromatography prior to mass spectrometry (MS) analysis using the following method. Nanoelectrospray capillary column tips were made in-house by using a P-100 laser puller (Sutter Instruments). The columns were packed with Zorbax SB-C18 stationary phase (Agilent) purchased in bulk (5-mm particles, with a 15-cm length and a 75-mm inner diameter). The reverse-phase gradient separation was performed by using water and acetonitrile (0.1% formic acid) as the mobile phases. The gradient consisted of 5% acetonitrile for 10 min followed by a gradient to 8% acetonitrile for 5 min, 35% acetonitrile for 113 min, 55% acetonitrile for 12 min, and 95% acetonitrile for 15 min. Data-dependent MS/MS data were obtained with an LTQ linear ion trap mass spectrometer using a home-built nanoelectrospray source at 2 kV at the tip. One MS spectrum was followed by 4 MS/MS scans on the most abundant ions after the application of the dynamic exclusion list. All MS/MS data were searched against the NCBI Mammalia (mammals) database using Mascot (version 2.3.02; Matrix Science, London, United Kingdom). Mascot searches were conducted using a peptide mass tolerance of 2.0 Da, a fragment ion mass tolerance of 0.80 Da, fixed modifications of carbamidomethylation (C), variable modifications of oxidation (M), an enzyme of trypsin, a maximum of one missed cleavage and decoy database with a peptide False Discovery Rate of 0.02. Proteins with a P < 0.05 (corresponding to a Mascot ion score greater than 57) were identified with two or more peptides and with a peptide False Discovery Rate of 0.02 considered at the 95% confidence level. The search did not detect glycosylated fragments.
